# Automated Large Vessel Occlusion Detection Software and Thrombectomy Treatment Times: A Cluster Randomized Clinical Trial

**DOI:** 10.1001/jamaneurol.2023.3206

**Published:** 2023-09-18

**Authors:** Juan Carlos Martinez-Gutierrez, Youngran Kim, Sergio Salazar-Marioni, Muhammad Bilal Tariq, Rania Abdelkhaleq, Arash Niktabe, Anjan N. Ballekere, Ananya S. Iyyangar, Mai Le, Hussain Azeem, Charles C. Miller, Jon E. Tyson, Sandi Shaw, Peri Smith, Mallory Cowan, Isabel Gonzales, Louise D. McCullough, Andrew D. Barreto, Luca Giancardo, Sunil A. Sheth

**Affiliations:** 1 Department of Neurosurgery, McGovern Medical School at UTHealth, Houston, Texas; 2 Center for Healthcare Data, School of Public Health, UTHealth, Houston, Texas; 3 Department of Neurology, McGovern Medical School at UTHealth, Houston, Texas; 4 Department of Cardiovascular and Thoracic Surgery, McGovern Medical School at UTHealth, Houston, Texas; 5 Department of Pediatrics, McGovern Medical School at UTHealth Houston, Texas; 6 Memorial Herman Hospital, Houston, Texas; 7 UTHealth School of Biomedical Informatics, Houston, Texas

## Abstract

**Importance:**

The benefit of endovascular stroke therapy (EVT) in large vessel occlusion (LVO) ischemic stroke is highly time dependent. Process improvements to accelerate in-hospital workflows are critical.

**Objective:**

To determine whether automated computed tomography (CT) angiogram interpretation coupled with secure group messaging can improve in-hospital EVT workflows.

**Design, Setting, and Participants:**

This cluster randomized stepped-wedge clinical trial took place from January 1, 2021, through February 27, 2022, at 4 comprehensive stroke centers (CSCs) in the greater Houston, Texas, area. All 443 participants with LVO stroke who presented through the emergency department were treated with EVT at the 4 CSCs. Exclusion criteria included patients presenting as transfers from an outside hospital (n = 158), in-hospital stroke (n = 39), and patients treated with EVT through randomization in a large core clinical trial (n = 3).

**Intervention:**

Artificial intelligence (AI)–enabled automated LVO detection from CT angiogram coupled with secure messaging was activated at the 4 CSCs in a random-stepped fashion. Once activated, clinicians and radiologists received real-time alerts to their mobile phones notifying them of possible LVO within minutes of CT imaging completion.

**Main Outcomes and Measures:**

Primary outcome was the effect of AI-enabled LVO detection on door-to-groin (DTG) time and was measured using a mixed-effects linear regression model, which included a random effect for cluster (CSC) and a fixed effect for exposure status (pre-AI vs post-AI). Secondary outcomes included time from hospital arrival to intravenous tissue plasminogen activator (IV tPA) bolus in eligible patients, time from initiation of CT scan to start of EVT, and hospital length of stay. In exploratory analysis, the study team evaluated the impact of AI implementation on 90-day modified Rankin Scale disability outcomes.

**Results:**

Among 243 patients who met inclusion criteria, 140 were treated during the unexposed period and 103 during the exposed period. Median age for the complete cohort was 70 (IQR, 58-79) years and 122 were female (50%). Median National Institutes of Health Stroke Scale score at presentation was 17 (IQR, 11-22) and the median DTG preexposure was 100 (IQR, 81-116) minutes. In mixed-effects linear regression, implementation of the AI algorithm was associated with a reduction in DTG time by 11.2 minutes (95% CI, −18.22 to −4.2). Time from CT scan initiation to EVT start fell by 9.8 minutes (95% CI, −16.9 to −2.6). There were no differences in IV tPA treatment times nor hospital length of stay. In multivariable logistic regression adjusted for age, National Institutes of Health Stroke scale score, and the Alberta Stroke Program Early CT Score, there was no difference in likelihood of functional independence (modified Rankin Scale score, 0-2; odds ratio, 1.3; 95% CI, 0.42-4.0).

**Conclusions and Relevance:**

Automated LVO detection coupled with secure mobile phone application-based communication improved in-hospital acute ischemic stroke workflows. Software implementation was associated with clinically meaningful reductions in EVT treatment times.

**Trial Registration:**

ClinicalTrials.gov Identifier: NCT05838456

## Key Points

**Question:**

Does implementation of automated large vessel occlusion detection software for acute stroke triage decrease time to endovascular thrombectomy initiation?

**Findings:**

In this cluster randomized trial including 243 patients treated with thrombectomy over a 1-year period, implementation of automated large vessel occlusion detection software led to a statistically significant reduction of 11 minutes in time to thrombectomy initiation.

**Meaning:**

Artificial intelligence-enabled automated large vessel occlusion detection software for stroke triage can improve thrombectomy treatment times.

## Teaser

This study attempts to determine whether automated computed tomography angiogram interpretation coupled with secure group messaging can improve in-hospital endovascular stroke therapy workflows.

## Introduction

Prompt endovascular thrombectomy (EVT) can dramatically improve outcomes in patients with large vessel occlusion (LVO) acute ischemic stroke (AIS); however, its efficacy is time dependent.^1,2,3,4,5^ In recent years, local and national efforts have focused on accelerating the time from hospital arrival to initiation of EVT and this metric has become a cornerstone for hospital stroke center certification.^6,7^ In the process of addressing this goal, key challenges have been identified and include clinicians and radiologists promptly recognizing the possibility of LVO AIS among the many patients they see and, subsequently, coordinating communication across the myriad care teams that prepare for and execute emergent EVT.^8,9^

Artificial intelligence (AI)–enabled LVO AIS screening coupled with Health Insurance Portability and Accountability Act-compliant group messaging may improve treatment times.^10,11,12,13,14^ These algorithms analyze computed tomography (CT) imaging in patients presenting with possible AIS and can determine the presence or absence of LVO on CT angiography (CTA) imaging within minutes, alerting radiologists and clinicians to the patient.^15,16^ The impact of such software has previously been evaluated in predominantly single-center pre-post observational studies, which have suggested improvements in hospital arrival to EVT initiation, total inpatient length of stay, and reduced time to transfer in patients with LVO AIS who present to non–EVT performing centers.^10,11,12,17^ However, because accelerating treatment times and specifically the time from hospital arrival to EVT initiation (ie, door-to-groin time [DTG]) is a vital part of AIS care and hospital accreditation, it is also the target of continuous in-hospital process improvement efforts.^6,7^ As such, pre-post observational studies can suffer from secular trends, in which the postintervention group would be confounded by the benefit of the ongoing process improvement effects.^18^

To determine the impact of AI-enabled automated LVO AIS detection and workflow enhancements on hospital arrival to EVT initiation, we performed a randomized stepped-wedge clinical study. We chose this design to overcome the impracticality of randomizing at the individual patient level but retaining a robust means to evaluate this intervention.^19^ We randomized 4 comprehensive stroke center (CSC) hospitals to initiate LVO detection software in predetermined stepped-time intervals and hypothesized that initiation of this intervention would result in a decrease in DTG time in patients with LVO AIS.

## Methods

### Data Availability and Trial Registration

Ethical approval was obtained from the UTHealth institutional review board and the requirement for patient consent was waived (HSC-MS-19-0630). This article was written and structured in compliance with Consolidated Standards of Reporting Trials (CONSORT) reporting guidelines, as well as guidelines specific for stepped-wedge trials.^20,21^

### Trial Design

We conducted a cluster randomized stepped-wedge clinical trial across 4 CSCs in the greater Houston-area from January 1, 2021, to February 27, 2022. This study design was chosen as it was not feasible to randomize at the individual patient level to AI software analysis. Because of the large number of health care professionals and the extreme acuity of decision-making involved in the care of patients with AIS, there was concern that any uncertainty on whether the communication platform could be used or whether alerts would be sent could negatively impact patient care. In addition, literature had previously been published demonstrating improvements in clinical care using this platform and the software had achieved US Food and Drug Administration clearance for this purpose.^17,22^ Furthermore, in 2021, the US Centers for Medicare and Medicaid Services granted a New Technology Add-On Payment for automated LVO detection. This designation provided additional payments on top of the established Medicare Severity Diagnosis-Related Group system and was awarded in recognition of the improved patient outcomes associated with implementation of this software.^23^ As such, there was hesitancy to withhold the software for a prolonged period after it had been activated. A stepped rollout and hospital-level cluster design was decided to be appropriate to both ensure efficient rollout across a large health system and for the purposes of a clinical trial. Of note, a stepped-wedge study design has been used to address a similar question of in-hospital process optimization in patients with EVT in a European cohort.^24^

Each CSC site corresponded to a single cluster and allocation to trial intervention was determined by the cluster. All patients treated with EVT during the trial period were included unless excluded for the reasons listed below in the Participants section. There was no blinding to allocation, as clinical care teams began to receive AI-enabled alerts once each cluster was activated and were aware of the activation time frames. Four steps were used, corresponding to the activation of 1 CSC per cluster at a time to the AI software. The duration of time between each step was determined a priori based on the volume of EVT procedures, as described in the sample size calculation section below. Periods were divided into pre-AI (unexposed), transition, and post-AI (exposed). The transition period lasted 2 weeks and corresponded to the period during which each CSC adjusted to the activation of the AI software (as described in the Intervention section below). In the final analysis, patients treated with EVT during the transition period were included in the pre-AI cohort, as described in the Statistical analysis section below. The order in which the clusters were activated was determined in a random fashion with 1 exception: the academic CSC was decided to be the final activation, as it was felt that additional time would be needed to prepare for this activation given the large number of residents and fellows involved in patient care at this CSC.

### Participants

Our trial population included patients with LVO AIS who presented to the emergency departments of 4 CSCs included in the trial and underwent imaging with CTA. In all 4 CSCs, imaging with noncontrast head CT followed immediately by CTA was performed in all patients presenting as code stroke evaluations throughout the study period as standard of care. Patients were included in the trial if they underwent emergent EVT for treatment of LVO AIS with occlusions of the internal carotid, middle cerebral, anterior cerebral, basilar, intracranial vertebral, or posterior cerebral arteries. Patients were excluded if they presented as in-hospital code stroke alerts or if they were transferred from non–EVT-performing centers to a CSC for EVT evaluation. These patients were excluded as the workflow for these patients is substantially different from the emergency department workflow, which was the primary focus of the intervention. In the case of patients transferred from other hospitals, the decision for EVT is usually made prior to transfer and the patients are brought directly to the angiography suite without repeating imaging. In addition, during the trial period, 2 of the CSCs participated in EVT trials of large core patients. Patients treated with EVT through randomization in one of these trials were also excluded, as these workflows also differed, due to the additional time required in consenting, enrolling and randomizing prior to EVT.

### Interventions

At the start of the trial period, noncontrast CT and CTA acquisition protocols were modified such that all imaging performed in the workup of patients presenting to the emergency departments for possible AIS was automatically transmitted to a cloud-based AI algorithm trained to detect LVO AIS from CTA (Viz.AI). This software package analyzes CTA images and arrives at a decision on the presence or absence of LVO within several minutes of receiving images. This software has been previously validated and shown to have high sensitivity and accuracy for LVO detection.^15^ Total processing time after image acquisition including transmission and software analysis was less than 5 minutes on average. The results of the algorithm are transmitted to a mobile phone application, which the clinical care team was required to download onto their phones, and arrived in the form of a pushed alert notification. Within the application, a mobile picture archiving and communication system (PACS) allowed users to verify imaging findings and a secure messaging platform allowed for communication by the entire care team. An overview of the changes in workflow associated with AI implementation can be seen in eFigure 1 in Supplement 1. While images were sent from each CSC to the software at the start of the study period, the alerts were not sent out until the preplanned activation date for each site. The corresponding patient images on the mobile PACS and the individual patient-level secure messaging platforms were also not available until activation. This design allowed for optimization of the imaging transmission protocols from the CT scanner to the cloud-based AI servers prior to each site’s activation but prevented exposure to the intervention prior to site activation. Following each cluster’s activation, clinical care teams began receiving alerts and had the ability to view images and discuss patient care within the mobile application. We measured software utilization by collecting the number of active users of the mobile application each week throughout the study period.

Software implementation strategies were identical across all sites. At the start of the study period, communication was sent to all the clinicians, radiologists, radiology technicians, and stroke care team members of the upcoming intervention. After the information technology security review had been completed, team members from the vendor then began working with CT technologists to modify CT acquisition protocols such that images would be sent at the time of acquisition to the cloud-based AI server. This process began at all sites at the start of the study period but increased in intensity closer to each sites’ activation. Weekly team meetings occurred between representatives from the vendor and a team consisting of each campus’s stroke coordinators and lead members from neurology, radiology, emergency medicine, neurointervention, and nursing to monitor progress. Two to 3 weeks prior to activation, a team of representatives from the vendor traveled in-person to the campus and hosted 2 days of education for the physicians and staff of the site. The purpose of these events was to remind the local team of the upcoming activation, demonstrate how to download and log in to the software, trouble shoot any other technical difficulties with software access, and send test images from the CT scanners to the cloud-based AI server to ensure that this process was functioning optimally. On the day of activation, additional notifications were sent to the hospital stroke team members to inform them of the change.

The 4 CSCs shared some similarities but also had differences. One CSC (CSC 4) was a major academic hub, with residents, fellows, and other trainees. Two other CSCs (CSCs 1 and 3) also had trainees but fewer in number. The four CSCs differed in annual AIS volume, as well as EVT volume as discussed below. During this study period, 2 CSCs (CSCs 1 and 4) also participated in large core EVT trials as discussed above. They were identical, however, with respect to electronic medical record system, stroke evaluation order sets, stroke evaluation protocols, institutional guidelines on EVT treatment eligibility, and image viewing systems. All CSCs had neurointensive care units and modern endovascular equipment with dedicated neurointerventional physician, nurse, and technologist teams. During daytime hours, all emergent stroke evaluations were performed by in-house physicians, including weekends. At nighttime, telemedicine was used. This system was consistent across all 4 CSCs. Some physicians rotated between the 4 sites, but most staff at each hospital were specific to that campus. For full study protocol, see Supplement 2.

### Outcomes

The primary outcome was the effect of AI-enabled LVO detection on DTG time. Secondary outcomes included time from hospital arrival to IV tPA bolus in eligible patients, time from initiation of initial noncontrast head CT scan to start of EVT, hospital length of stay, and rates of substantial reperfusion after EVT (thrombolysis in cerebral infarction score of 2b/3). As noted above, CTA was performed immediately after initial noncontrast head CT in all patients. Safety outcomes included the rate of mortality, any intracerebral hemorrhage following EVT, and rates of symptomatic intracerebral hemorrhage, which were defined as parenchymal hematoma type 2 with associated worsening of 4 points or more on the National Institutes of Health Stroke Scale (NIHSS).^25,26^ We performed an exploratory analysis to study the effect of our intervention on functional independence at discharge and 90 days (modified Rankin score, 0-2 vs 3-6). We also evaluated the effect of time (ie, days after initiation of the trial) on DTG time across the study period to evaluate for secular trends.

### Sample Size Calculation and Determination of Length of Time for Clusters

We a priori determined the length of the trial period and each step with several considerations in mind. First, we wanted to ensure sufficient sample within each step; however, we also wanted to keep the overall trial period brief enough to minimize the effect of secular trends on the primary outcome. The total volume across all 4 CSCs is about 380 per year and we estimated about 200 of these procedures would meet eligibility criteria based on a review of the previous year’s data. The CSC with the lowest annual volume performs roughly 50 cases per year and we estimated about 30 such cases would meet eligibility criteria based on a review of the previous year’s data. From these considerations, we chose an overall trial period of 14 months, estimating a total sample size of approximately 225. We chose a slightly longer first period to ensure adequate sample size of the pre-AI cohort; a period of 4 months was chosen to ensure that the CSC with the lowest volume would have at least 10 cases during this period, in the event that it was the first hospital to be activated. The subsequent steps were then timed to take place 6 weeks apart, with a period of approximately 3 months at the end of the trial to ensure adequate capture of the post-AI cohort.

### Statistical Analysis

The primary outcome was analyzed using a mixed-effects linear regression model, which included a random effect for cluster (CSC) and a fixed effect for exposure status (pre-AI vs post-AI). The model was adjusted for age, sex, and NIHSS. Secondary outcomes including time from emergency department arrival to IV tPA treatment, hospital length of stay, and time from CT scan initiation to EVT start were analyzed using the same method, adjusted for age, sex, and NIHSS. In sensitivity analysis, we also adjusted the model for time from trial initiation (in months) to more explicitly account for secular trends, as has been recommended for the analysis of stepped-wedge study designs.^27^ In further sensitivity analysis, we repeated the analysis in the subset of patients with large vessel occlusions of the proximal anterior circulation alone (ie, internal carotid artery or middle cerebral artery) and on the subset of patients that presented in the early time window (within 6 hours from last known well). An exploratory analysis on the likelihood for functional independence (modified Rankin Scale [mRS], 0-2) at 90 days was performed using logistic regression adjusted for age, NIHSS, and Alberta Stroke Programme Early CT Score (ASPECTS). The effect of time from study start (in months) on DTG was determined by linear regression in the entire cohort and separately in the subset of patients treated in the pre-AI and post-AI periods. Univariable comparisons between categorical variables were performed using χ^2^and between continuous variables with the Wilcoxon rank sum test. We calculated the Rapid Arterial Occlusion Evaluation score from the subcomponent NIHSS scores, consistent with prior literature that has shown equivalence between these 2 approaches because multiple prehospital scales are used in the region.^28^ Analyses were performed using STATA version 17 (StataCorp) and Prism version 9 (GraphPad).

## Results

**Figure 1.**
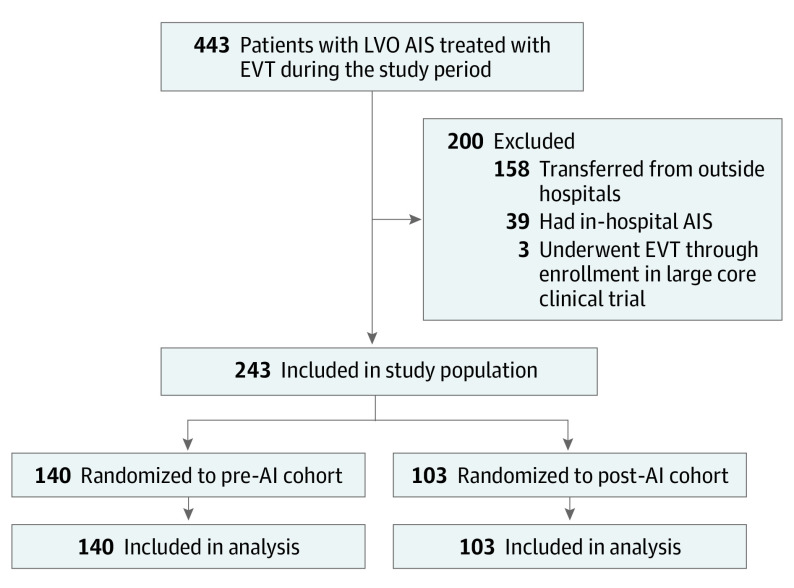
Flow Diagram Showing Enrollment, Allocation, and Follow-Up Population Analyzed AI indicates artificial intelligence; AIS, acute ischemic stroke; EVT, endovascular stroke therapy; LVO, large vessel occlusion.

**Figure 2.**
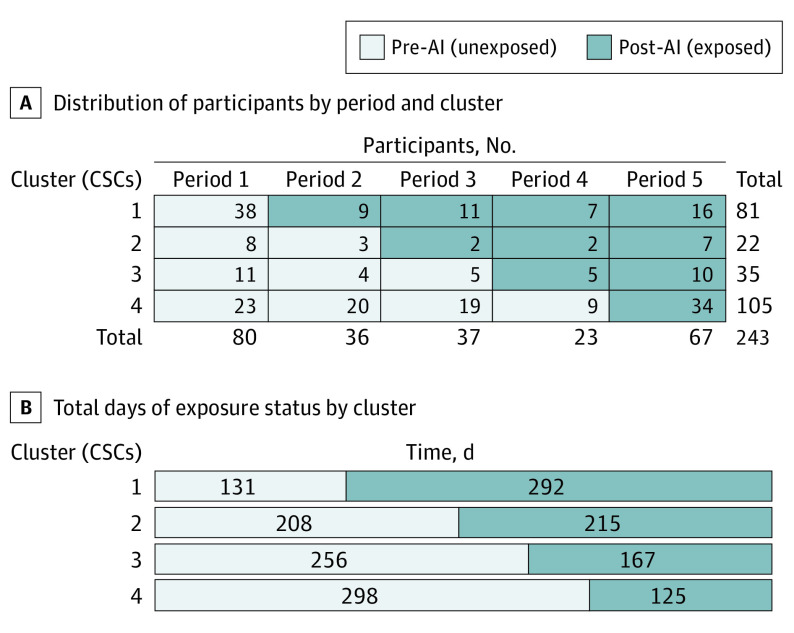
Structure of the Stepped-Wedge Design A, Distribution of participants by period and cluster with unexposed and exposed sections demarcated. B, Total days of exposed and unexposed status by cluster. AI indicates artificial intelligence; CSC, comprehensive stroke center.

Among 243 patients who met inclusion criteria (Figure 1), 131 were treated during the unexposed period, 103 during the exposed period, and 9 patients in the transition period. All patients were analyzed according to their allocated cluster and activation dates were on time as planned throughout the trial (Figure 2). Note that Figure 2 depicts the clusters as used in the primary analysis, and as such, the transition periods are included in the unexposed cohorts. As shown in Table 1, median age for the complete cohort was 70 (IQR, 58-79) years and 122 patients were female (50%). Median NIHSS score at presentation was 17 (IQR, 11-22) and the median time from last known well to arrival was 132 (IQR, 61-498) minutes. Pre-AI and post-AI cohorts were largely balanced apart from a slight increase in ASPECTS in the post-AI cohort.

**Table 1.** Patient Demographics

Characteristic	Total cohort (n = 243), No. (%)	Pre-AI (n = 140), No. (%)	Post-AI (n = 103), No. (%)	*P* value
Age, y, median (IQR)	70 (58-79)	69.5 (58.5-78)	71 (57-79)	.68
Sex				
Female	122 (50)	73 (52)	49 (48)	.48
Male	121 (50)	67 (48)	54 (52)	
Race and ethnicity^a^				
African American	69 (28)	42 (30)	27 (26)	.80
Asian	13 (5)	7 (5)	6 (6)	
Hispanic	41 (17)	25 (18)	16 (16)	
White	108 (44)	58 (41)	50 (49)	
Other^b^	12 (5)	8 (6)	4 (4)	
Prestroke mRS				
0	164 (71)	97 (72)	67 (68)	.15
1	26 (11)	16 (12)	10 (10)	
2	19 (8)	13 (10)	6 (6)	
3	22 (10)	8 (6)	14 (14)	
Medical history				
Prior stroke	43 (18)	24 (17)	19 (18)	.79
Prior TIA	16 (7)	11 (8)	5 (5)	.35
HTN	182 (75)	107 (76)	75 (73)	.52
HLD	88 (36)	55 (39)	33 (32)	.25
Atrial fibrillation	71 (29)	41 (29)	30 (29)	.98
Diabetes	69 (28)	46 (33)	23 (22)	.07
Smoking	51 (21)	28 (20)	23 (22)	.66
CHF	27 (11)	15 (11)	12 (12)	.82
Last known well to hospital arrival, min, median (IQR)	132 (61-498)	131.5 (61-472)	147 (65-569)	.60
NIHSS score	17 (11-22)	17 (11-23)	16 (11-21)	.19
RACE score ≥5	187 (77)	110 (79)	77 (75)	.49
CT ASPECTS	9 (7-10)	9 (7-10)	10 (8-10)	.04
IV tPA	111 (46)	63 (45)	48 (47)	.80
Occlusion location				
Internal carotid artery	42 (17)	29 (21)	13 (13)	.11
M1 middle cerebral artery	111 (46)	56 (40)	55 (53)	
M2 middle cerebral artery	51 (21)	29 (21)	22 (21)	
Basilar artery	11 (5)	9 (6)	2 (2)	
Cervical internal carotid artery and tandem intracranial	9 (4)	4 (3)	5 (5)	
Posterior cerebral artery	5 (2)	2 (1)	3 (3)	
Vertebral artery	3 (1)	3 (2)	0 (0)	
Other	11 (5)	8 (6)	3 (3)	

Abbrevations: AI, artificial intelligence; ASPECTS, Alberta Stroke Programme Early CT Score; CHF, congestive heart failure; CT, computed tomography; HLD, hyperlipidemia; HTN, hypertension; IV tPA, intravenous tissue plasminogen activator; mRS, modified Rankin Scale score; NIHSS, National Institutes of Health Stroke Scale; RACE, rapid arterial occlusion evaluation; TIA, transient ischemic attack.

^a^ Race and ethnicity were self-reported.

^b^ Includes Native American and not specified.

CSC activation was associated with a sharp and sustained increase in software utilization. As shown in eFigure 2 in Supplement 1, there was minimal or no activity on the mobile application as measured by the total number of active users prior to the site activation apart from a small amount of testing activity during the onboarding process as described above. After activation, there was a sharp rise in software utilization, and this utilization was maintained throughout the rest of the study period. This pattern was consistent across all 4 sites.

The number of patients and duration of time for each step and period can be seen in Figure 2. As shown in Table 2 in univariable analysis, the study team observed a reduction in DTG time of 12 minutes in the pre-AI to post-AI cohort. There was also a reduction in time from initiation of CT scan to the start of EVT. The study team did not observe any differences in IV tPA treatment times, length of stay, rates of functional independence, or safety outcomes apart from mortality, which decreased after the AI intervention.

**Table 2.** Clinical Outcomes and Time Metrics

Characteristic	Total cohort (n = 243), No. (%)	Pre-AI (n = 140), No. (%)	Post-AI (n = 103), No. (%)	*P* value
DTG time, min (IQR)	97 (75-113)	100 (81-116)	88 (65-110)	.002
Hospital arrival to IV tPA bolus time, min (IQR)	30 (21-41)	30 (22-44)	28 (21-36)	.14
Initiation of CT scan to EVT start time, min (IQR)	80 (60-96)	85 (68-99)	72 (55-90)	<.001
Length of stay, d (IQR)	7 (4-11)	7 (4-12)	6 (3-10)	.11
TICI 2b/3 reperfusion	219 (90)	124 (89)	95 (92)	.34
Safety outcomes				
Symptomatic ICH	9 (4)	7 (5)	2 (2)	.21
Any ICH	43 (18)	24 (17)	19 (18)	.81
Mortality	57 (23)	44 (31)	13 (13)	<.001

Abbreviations: AI, artificial intelligence; CT, computed tomography; DTG, door to groin; EVT, endovascular stroke therapy; ICH, intracerebral hemorrhage; IV tPA, intravenous tissue plasminogen activator; TICI, thrombolysis in cerebral infarction.

Table 3 presents the primary and secondary outcomes. In mixed-effects linear regression, implementation of the LVO detection AI algorithm resulted in a reduction in DTG time by 11.2 minutes. Time from CT scan initiation to EVT start fell by 9.8 minutes. There were no differences in IV tPA treatment times nor hospital length of stay. Sensitivity analysis, in which time from study onset was measured in months, was also included as a covariable in the regression, the improvement in DTG time was unchanged (−11.3 minutes; 95% CI, −22.1 to −0.52). In further sensitivity analysis, the improvement in DTG was largely unchanged in the cohort of patients with anterior circulation LVO (−12.9 minutes; 95% CI, −20.3 to −5.5) and those that presented within the first 6 hours from last known well (−13.0 minutes; 95% CI, −21.4 to −4.5). In the subset of patients treated with IV tPA, the DTG reduction was less and did not achieve statistical significance (−6.6 minutes; 95% CI, −19.2 to 3.0). On the other hand, the reduction in DTG was more robust in the subset of patients who did not receive IV tPA (−14.3 minutes; 95% CI, −24.5 to −4.2). Additionally, the effect was maintained in the subset of patients with NIHSS more than 10 (−12.3 minutes; 95% CI, −19.6 to −4.9]).

**Table 3.** Mixed-Model Stepped-Wedge Outcomes^a^

Characteristic	Coefficient (95% CI)	*P *value
Primary outcome		
DTG time, min	−11.2 (−18.22 to −4.2)	<.01
Secondary outcomes		
Initiation of CT scan to EVT start time, min	−9.8 (−16.9 to −2.6)	<.01
Hospital arrival to IV tPA bolus time, min	−3.5 (−9.1 to 2.2)	.23
Length of stay, d	−0.4 (−2.6 to 1.7)	.72

Abbrevations: CT, computed tomography; DTG, door to groin; EVT, endovascular stroke therapy; IV tPA, intravenous tissue plasminogen activator.

^a^ Regressions adjusted for age, sex, and National Institutes of Health Stroke Scale score.

The study team also examined the effect of time during the trial period on DTG. As shown in eFigure 3 in Supplement 1, for the entire cohort there was a reduction in DTG time from the start of the study to the conclusion (coefficient −0.03 [minutes per day]; *P* < .05). Given that this analysis would be confounded by the AI intervention, the study team then separately analyzed patients in the pre-AI cohorts (eFigure 3B in Supplement 1) and post-AI cohorts (eFigure 3C in Supplement 1). There was no difference in DTG during the trial period in the pre-AI cohort (coefficient −0.007 [minutes per day]; *P* = .80) nor the post-AI cohort (coefficient −0.01 [minutes per day]; *P* = .80).

In exploratory analyses on the impact of the software intervention on clinical outcomes, rates of functional independence at 90 days (mRS, 0-2) were similar in univariable comparisons of the pre-AI and post-AI cohorts (32% vs 42%, pre-AI vs post-AI; *P* = .47). In multivariable logistic regression adjusted for age, NIHSS and ASPECTS, there was no observe a difference in likelihood of 90-day disability (mRS, 0-2) in the post-AI cohort relative to pre-AI (odds ratio, 1.3; 95% CI, 0.42-4.0). Similarly, there were no differences in rates of good functional outcomes at discharge defined as mRS 0-2 (28% vs 41%, pre-AI vs post-AI; *P* = .17).

## Discussion

In this prospective cluster randomized trial of nearly 250 patients with LVO AIS presenting to emergency departments and treated with EVT across 4 CSCs, activation of AI-enabled LVO alerts resulted in an 11-minute reduction in DTG time. Time from CT initiation to EVT start fell by a similar amount and rates of mortality fell by nearly 60%. We did not observe significant differences in hospital length of stay, discharge, or 90-day functional outcomes.

Prior studies have examined the effect of automated LVO detection on in-hospital quality metrics, as well as clinical outcomes. Several studies have found statistically significant reductions in DTG time and one study identified a reduction in hospital length of stay.^29,30^ These prior studies have been limited by several factors, which include small sample sizes, single-hospital data, and heterogenous populations that include both transfer patients as well as direct emergency department presentations. In addition, none of these prior studies have been able to account for secular changes in DTG time. The DTG metric is a key component of CSC certification requirements, and as such it is consistently targeted by national quality improvement initiatives and continuous efforts at local levels.^7^ For this reason, it is likely that a pre-post model will be confounded by alternative efforts to address this endpoint. In this trial, we did not observe substantial reductions in DTG time in the pre-AI cohort, possibly because the trial period was much shorter than previous studies examining this software.^11^

There are several mechanisms by which DTG reduction could have occurred in this study. First, the automated LVO alert arrives within minutes of CTA completion, far earlier than human interpretation, and in many cases even before the CTA images were available for review on the clinical PACS. In addition, the process for screening, evaluating, and ultimately treating a patient with EVT can be highly complicated with many different care teams that need to work in synchrony. Prior studies have shown that organizing this process into a parallel workflow, rather than serial, can improve DTG times.^8,9^ By incorporating all the relevant team members into a single communication platform, parallel processing may become easier. Our finding that time from CT initiation to EVT start fell by approximately 10 minutes, which is much of the time reduction seen in DTG, supports this possibility. This phase of the in-hospital EVT workflow is often the most complicated and likely benefited from transparent and efficient communication across the myriad involved clinical services including anesthesia, imaging, patient transport, neurology, neurointervention, and others. Additionally, with implementation of this software, LVO alerts were now widely available and on an easily accessible, real-time record. It is possible that the knowledge that they could be observed changed care teams’ behavior. This effect has been seen in another study, wherein requiring IV tPA treatment times to be reported widely by pager in real time reduced treatment times.^31^ A similar Hawthorne effect may have contributed to the DTG times associated with this LVO detection software.^32^

We did not observe any improvements in 90-day disability outcomes or in length of stay associated with the intervention. Likelihood of good outcome is known to deteriorate with delays in EVT, and in some series, statistically significant improvements in mRS could be seen with 15-minute accelerations in care.^3,5^ These studies, however, were derived from clinical trials with very strictly defined inclusion criteria that include limits on age, NIHSS, time from onset to treatment, ASPECTS, and many other parameters. In a cohort as heterogeneous as this one, in which nearly all patients with EVT presenting through the emergency department were included, it is unlikely that our sample size would provide sufficient power to detect a difference in clinical outcomes with an 11-minute acceleration in care. We did observe a reduced rates of mortality although the explanation is not immediately clear. Prior studies have identified NIHSS, ICA occlusion location, and symptomatic hemorrhage rates as predictors of mortality.^33,34^ In this study, we did not identify any differences in NIHSS, but there were nonsignificant lower rates of ICA occlusion location and rate of symptomatic intracerebral hemorrhage in the post-AI cohort. It is possible that these factors played a role in the reduced rates of mortality.

### Limitations

This study suffers from a few limitations that may limit generalizability. First, while 4 CSCs were included in the analysis, they are all a part of a single health care system, unified by a single electronic medical record and the same overarching stroke process workflows. Some physicians also rotated between several of the CSCs, resulting in a level of consistency across the hospitals. Also, the preintervention DTG times were near 100 minutes. It is possible that the effect size may be different in other settings, in which the preintervention DTG time is substantially longer or shorter. However, the 4 CSCs included in this study covered a wide range of hospital types, from a high-volume tertiary referral academic medical center to lower volume community-based hospitals without physician trainees. In addition, this value of 100 minutes for DTG is highly consistent with those seen in a large nationwide cohort of 231 hospitals and nearly 14 500 patients, in which median DTG time for patients arriving through the emergency department was 100 (IQR, 78-127) minutes and in another similar but more recent cohort of nearly 54 000 patients across 697 sites in the US, in which median DTG time was 108 (IQR, 66-164) minutes.^35,36^ Another limitation of the trial may be the unblinded nature of the intervention. On the other hand, blinding to LVO alert and communication platform would not be possible, and as discussed above, some of the efficacy of the intervention may derive from the knowledge that patient alerts and care team discussions were more widely viewable.

## Conclusions

In this cluster randomized stepped-wedge clinical trial, automated LVO detection coupled with secure phone application-based communication improved in-hospital AIS workflows. Software implementation was associated with clinically meaningful reductions in EVT treatment times.

## Notes

Supplement 1. eTable. Dates of activation and transition periods by cluster eFigure 1. Workflow diagrams demonstrating the intervention evaluated in this clinical trial eFigure 2. Number of active users on the mobile application throughout the study period per week by individual cluster/CSC eFigure 3. Linear regressions of the effect of time on door to groin time

Supplement 2. Study protocol

Supplement 3. Data sharing statement
