# Supplementary material for: Automated Large Vessel Occlusion Detection Software and Thrombectomy Treatment Times: A Cluster Randomized Clinical Trial
Source: JAMA Neurol. 2023 Sep 18;80(11):1182–90. doi: 10.1001/jamaneurol.2023.3206 (PMC10507590; doi:10.1001/jamaneurol.2023.3206)
Supplement: Supplement 1. — eTable. Dates of activation and transition periods by cluster eFigure 1. Workflow diagrams demonstrating the intervention evaluated in this clinical trial eFigure 2. Number of active users on the mobile application throughout the study period per week by individual cluster/CSC eFigure 3. Linear regressions of the effect of time on door to groin time [file jamaneurol-e233206-s001.pdf]

Supplemental Online Content

Martinez-Gutierrez JC, Kim Y, Salazar-Marioni S, et al. Diagnostic testing for celiac disease among patients with abdominal symptoms in primary care: a systematic review. *JAMA Neurol*. Published online September 18, 2023.
doi:10.1001/jamaneurol.2023.3206

eTable. Dates of activation and transition periods by cluster
eFigure 1. Workflow diagrams demonstrating the intervention evaluated in this clinical trial
eFigure 2. Number of active users on the mobile application throughout the study period per week by individual cluster/CSC
eFigure 3. Linear regressions of the effect of time on door to groin time

This supplemental material has been provided by the authors to give readers additional information about their work.

© 2023 Martinez-Gutierrez JC et al. *JAMA Neurol.*

S. Figure 1

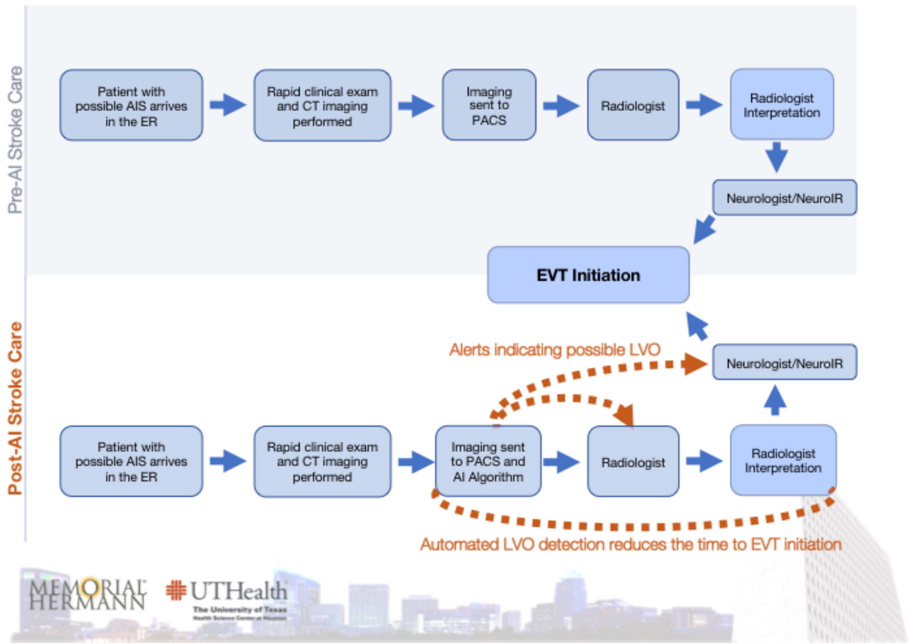

© 2023 Martinez-Gutierrez JC et al. *JAMA Neurol.*

S. Figure 2

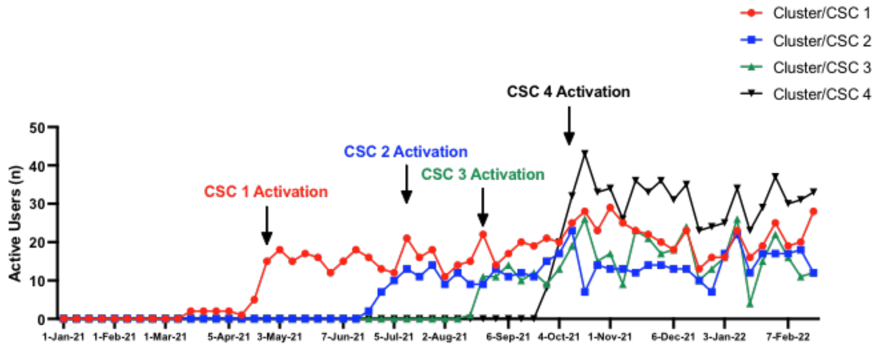

© 2023 Martinez-Gutierrez JC et al. *JAMA Neurol.*

S. Figure 3

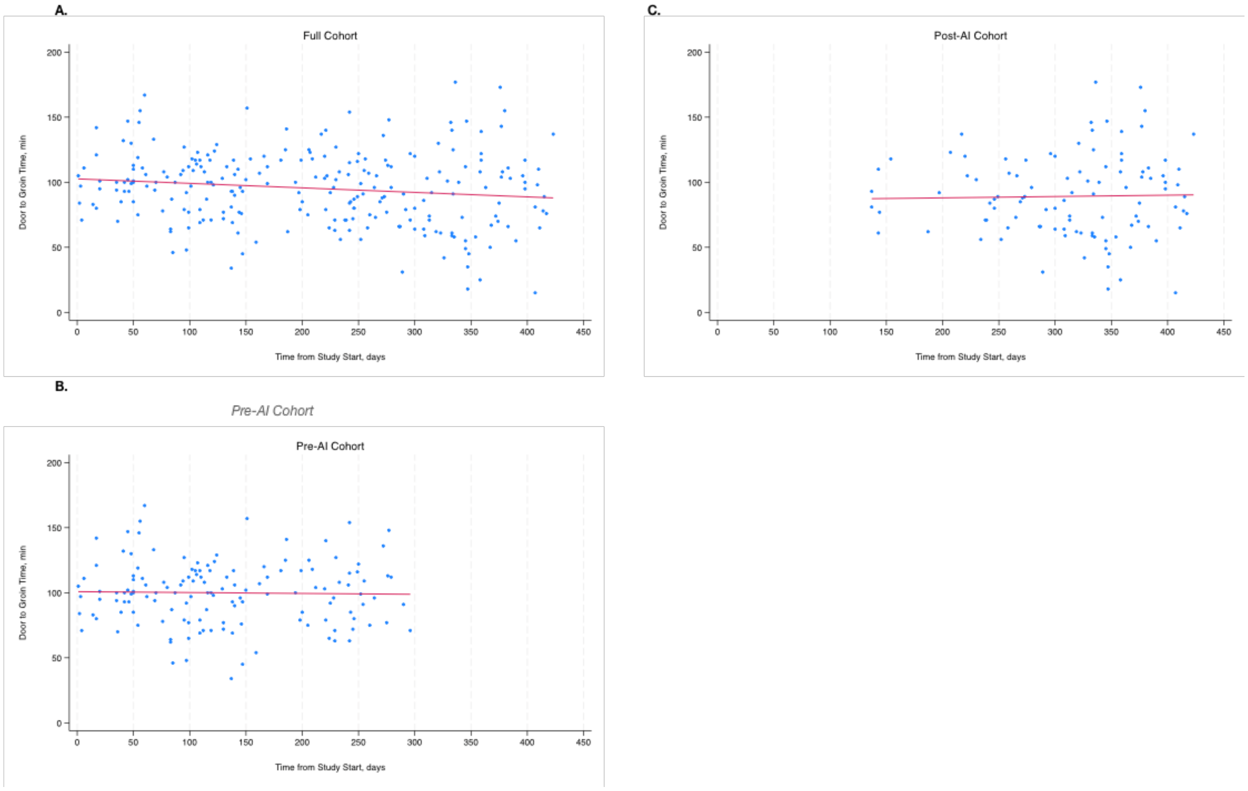

© 2023 Martinez-Gutierrez JC et al. *JAMA Neurol.*

S. Table 1

Steps	Cluster/CSC	Start Date	Activation Date	Exposure Date (after transition period)	End Date
**1**	1	1/1/2021	4/28/2021	5/12/2021	2/28/2022
**2**	2	1/1/2021	7/14/2021	7/28/2021	2/28/2022
**3**	3	1/1/2021	8/31/2021	9/14/2021	2/28/2022
**4**	4	1/1/2021	10/12/2021	10/26/2021	2/28/2022

© 2023 Martinez-Gutierrez JC et al. *JAMA Neurol.*
