# Supplementary material for: Automated Large Vessel Occlusion Detection Software and Thrombectomy Treatment Times: A Cluster Randomized Clinical Trial
Source: JAMA Neurol. 2023 Sep 18;80(11):1182–90. doi: 10.1001/jamaneurol.2023.3206 (PMC10507590; doi:10.1001/jamaneurol.2023.3206)
Supplement: Supplement 2. — Study protocol [file jamaneurol-e233206-s002.pdf]

**Protocol Title:**	Deep Learning Enabled Endovascular Stroke Therapy Screening in Community Hospitals
**Protocol Version/Date:**	3.0, 01/16/2023
**Principal Investigator:**	Sunil A. Sheth, MD, Luca Giancardo, PhD
**Co-Investigators:**	Youngran Kim, MD
**Key Study Personnel:**	Sergio A. Salazar Marioni
**Population:**	5,000 subjects
**Number of Sites:**	4 Sites<br>Memorial Hermann Hospital – TMC<br>Memorial Hermann Hospital – Memorial City<br>Memorial Hermann Hospital – Southwest<br>Memorial Hermann Hospital – Woodlands
**Study Duration:**	July 15th 2019 – March 01st, 2026
**Subject Duration:**	Not applicable

Acute Ischemic Stroke (AIS) is the leading cause of long-term disability in the US, and every minute of delay from onset to treatment reduces the likelihood of good clinical outcome. As a result of our ongoing Comprehensive Stroke Center (CSC) quality improvement efforts, we have identified a key delay: time from the completion of computed tomography (CT) angiography of the head and neck to interpretation in the setting of AIS care. Motivated by this unmet need in clinical care, and in partnership with the UTHealth School of Biomedical Informatics, we recently developed a machine-learning algorithm to automate the interpretation of CT angiography (CTA) for the presence or absence of large vessel occlusion (LVO) and other stroke relevant information such as estimated stroke core size. We propose a prospective, cluster- randomized study to determine whether incorporating our automated machine learning (ML) algorithms to AIS care pathways reduces the time from patient ER arrival to imaging interpretation by sending automated alerts of its findings to physicians on the stroke care team. Physicians will ultimately decide if patients are eligible for thrombectomy, and all patients will receive the standard of care following current guidelines. This strategy is expected to help improve patient stratification procedures for endovascular thrombectomy by using commonly available data. Our unique research team consisting of machine-learning and AIS imaging experts will take advantage of the rich and diverse talents across the UTHealth Campuses and Memorial Hermann Hospital System.

UTHealth Houston
IRB NUMBER: HSC-MS-19-0630
IRB APPROVAL DATE: 02/09/2023

Background Information

Acute Ischemic Stroke (AIS) is the leading cause of long-term disability in the US adult population with nearly 800,000 new patients affected by stroke annually in the US, and with Large Vessel Occlusion (LVO), a subtype of AIS that could require endovascular stroke therapy (EST), disproportionately contributing to stroke-related dependence and death.[1,2] The last four years, however, have borne witness to rapid changes in care for patients with LVO AIS. In 2015, five randomized clinical trials established EST as one of the most significant advances in modern medicine.[3-7] Compared to patients treated with medical management alone, LVO AIS patients treated with EST were nearly 2.5 fold more likely to achieve good neurological outcomes (number needed to treat = 3), a treatment effect size rivaling that of antibiotics to treat acute bacterial infections.[8,9] Yet these advances have revealed shortcomings in our AIS systems of care, as we are now faced with a great number of patients that must be triaged with an increasingly complex evaluation that must be performed in a shorter time.

A known dogma is that time is brain in AIS. Every minute after an occlusion of the cerebral vasculature 2 million neurons die, and key quality metrics in AIS revolve around reducing the time from when the patient arrives to the hospital to when treatment is initiated.[13] While we now know from the results of pivotal randomized clinical trials that EST, which re-opens occluded cerebral vessels through catheter-based procedures, can dramatically improve patient outcomes following AIS, these outcomes are still highly time-dependent. [10,11] This challenge is particularly relevant in the non-Texas Medical Center MH campuses, where delays after imaging arise for two reasons. First, Neurologists caring for patients in these settings are not comfortable interpreting the imaging studies to determine the presence or absence of LVO without relying on a Radiologist interpretation, thereby adding an additional step to the care pathway. Second, for Neurologists evaluating these patients remotely through Telemedicine, the imaging studies may not be completed at the time of the initial evaluation, and the requirement for the physician to remember to check periodically to see when the imaging study is complete can lead to fall outs, particularly when they may have moved on to care for another patient.

Through our continuous process of quality improvement, we recently conducted an analysis of delays in AIS treatment. Among 833 patients with AIS, median time from ER arrival to CTA interpretation for presence or absence of LVO was 65 minutes [IQR 41 - 127]. In order to comply with our Joint Commission requirements for Comprehensive Stroke Center, the EST procedure must be initiated within 90 minutes from patient arrival to the ER, and given the multiple steps that must occur after the imaging result and before EST initiation, this time interval from ER arrival to imaging result was identified as a weak point in the process.

Motivated by this unmet need in clinical care, and in partnership with the UTHealth School of Biomedical Informatics, we recently developed a machine-learning algorithm to automate the interpretation of CTA for the presence or absence of LVO. This novel deep neural network architecture, named DeepSymNet, was trained on over 400 CTA studies, using the radiology report and expert readers interpretations as gold standard, and can nearly instantaneously analyze images for LVO.[12] We then validated this algorithm on a fully external dataset of 126 patients, and found that it can perform this task with very high discrimination (Figure 1).

UTHealth Houston
IRB NUMBER: HSC-MS-19-0630
IRB APPROVAL DATE: 02/09/2023

Given these results, we propose a prospective, cluster-randomized study to determine whether AIS care pathways incorporating image based ML algorithms reduces the time from patient ER arrival to imaging interpretation (Figure 2). Our unique research team consisting of machine-learning and AIS imaging experts will take advantage of the rich and diverse talents across the UTHealth Campuses and the Memorial Hermann Hospital System. Given our preliminary findings, we hypothesize that integration of these approaches into the AIS care pathway will reduce delays from CTA acquisition to interpretation, without requiring any change in existing CT hardware or acquisition protocols.

Objectives

The expected outcome is the creation of a state-of-the-art infrastructure for deploying artificial intelligence algorithms in the clinical practice and a thorough evaluation of a potential paradigm shifting approach for a measurable and sustainable quality improvement of stroke care.

Study Design

To test the previously mentioned hypotheses, we propose a multi-center, stepped wedge cluster-randomized study that will include patients across 4 hospitals in the Memorial Hermann/UTHealth stroke system of care in the greater Houston area. The software will be randomly enabled at each of 4 comprehensive stroke center hospitals to evaluate the impact of this software on primary and secondary outcomes. The primary outcome will be the time from patient ER arrival to initiation of EST ("door-to-groin" time). Secondary outcomes include the number of patients treated with EST and 90-day disability outcomes.

Study Population

The alert system will be enabled for all CT angiography imaging studies for participating Hospitals. A valid sample will be one that satisfy the following criteria:

Inclusion Criteria

1. Male or Female, 18 years of age or older.
2. Patients who present to the emergency department with signs and/or symptoms concerning for acute ischemic stroke.
3. Patients who undergo CT angiography imaging
4. Patients determined to have a large vessel occlusion acute ischemic stroke. This determination will be made based on official radiology report for the CT angiography imaging.

UTHealth Houston
IRB NUMBER: HSC-MS-19-0630
IRB APPROVAL DATE: 02/09/2023

Exclusion Criteria

1. Patients with incomplete data on the electronic medical record.

Study Procedures

ML Algorithms Integration

Custom ML software (Viz.AI) will be installed on a server and receive data from the centralized PACS system. This software has been purchased by the MH hospital system for commercial use. The system will then send alerts to the care team through notifications pushed through the Viz.AI app present on the physician, nurse and technologist's mobile phone. Diagnosis and treatment decisions will be based on the clinical evaluation and review of the images by the treating physician, as per routine standard of care. This study will not interfere with routine clinical work. Data capture will be performed after treatment decisions and care have been delivered.

Clinical Data Acquisition

Clinical data will be collected at each of the participating sites as a part of our ongoing Comprehensive Stroke System quality improvement protocols. All other clinical and outcome data will be collected through electronic medical record review. Captured data will include patient demographics, past medical history, imaging utilization and imaging findings, time intervals (i.e. time from last known well to presentation, time from door to imaging, time from door to IV tPA, time from door to groin puncture), procedural outcomes (i.e. TICI scores, hemorrhage rates), discharge clinical outcomes, and in patients who received EST or IV tPA, 90-day disability outcomes. These data are routinely captured per standard-of-care at our Comprehensive Stroke System and will be used for analysis as a part of this study.

Ethics

Being an observational project, there will be no interventions, investigations, tests, drugs, and/or interviews administered to patients. We do not expect any research-related adverse events. All personal health information from patients will be kept confidential and in compliance with HIPAA regulations. This site is seeking waver of consent for this observational study involving electronic medical chart review and analysis of de-identified CTA images.

Risks and benefits for the human subjects

Risks

In this study, ML algorithms are not used for diagnosis, cure, mitigation, treatment or prevention as it only sends alerts. All diagnosis and treatment decisions are still based on the clinical evaluations and radiology reports. As such, we do not foresee that this study poses more than minimal risk encountered during the routine stroke care.

Benefits

We do not foresee any immediate benefits for the human subjects involved. However, if successful, this study could enable a faster time-to-treatment for stroke patients.

Data handling and record keeping

UTHealth Houston
IRB NUMBER: HSC-MS-19-0630
IRB APPROVAL DATE: 02/09/2023

Imaging Data

The server running the ML algorithm will hosted by Viz.AI. This server and imaging transfer protocol have been reviewed by MH IT and found to fall within acceptable security parameters. The Viz.AI software is a commercial product, and its usage in this study will fall within the boundaries of its intended use as a commercial product and the contract with MH hospital.

Clinical Data

All the clinical numerical variables collected for this study will be anonymized and stored in a central RedCap database inside the UTHealth network. All users of this database will comply with HIPAA regulations and with Good Clinical Practice for the handling of electronic clinical data. Only the study key personnel will have access to the project data.

Each subject will be given a unique subject identification which will be linked to the subject's MRN for prospective data collection. If data is to be analyzed, no patient identifier will be included to ensure no patient can be tracked or identified. Thus, analysis will solely be performed on de-identified data. No paper records will be kept. To ensure reproducibility of the finding, the UTHealth RedCap will continue to store the data after the completion of the study and until IT resources will allow to maintain the machine updated and protected. If this is not possible, all the data containing Protected Health Information (PHI) will be destroyed.

Data Sharing

In order to allow for a broader reach for our finding and scientific publications, we might have to share part of the data for scientific non-commercial purposes. In this case, we will strip the imaging and clinical data of any PHI information.

Publication Plan

Results based on data gathered from this this study may serve to elaborate abstract submissions or manuscripts for peer-reviewed journals. Research publications will not include any patient-specific information or identifiers.

References

1. Benjamin, E.J. *et al.* Heart Disease and Stroke Statistics-2018 Update: A Report From the American Heart Association. *Circulation* **137,** e67–e492 (2018).
2. Malhotra, K., Gornbein, J. & Saver, J. L. Ischemic Strokes Due to Large-Vessel Occlusions Contribute Disproportionately to Stroke-Related Dependence and Death: A Review. *Front Neurol* **8,** 651 (2017).
3. Berkhemer, O. A. *et al.* A randomized trial of intraarterial treatment for acute ischemic stroke. *N. Engl. J. Med.* **372,** 11–20 (2015).
4. Campbell, B. C. V. *et al.* Endovascular therapy for ischemic stroke with perfusion-imaging selection. *N. Engl. J. Med.* **372,** 1009–1018 (2015).
5. Goyal, M. *et al.* Randomized assessment of rapid endovascular treatment of ischemic stroke. *N. Engl. J. Med.* **372,** 1019–1030 (2015).
6. Saver, J. L. *et al.* Stent-retriever thrombectomy after intravenous t-PA vs. t-PA alone in stroke. *N. Engl. J. Med.* **372,** 2285–2295 (2015).
7. Jovin, T. G. *et al.* Thrombectomy within 8 Hours after Symptom Onset in Ischemic Stroke. *N. Engl. J. Med.* 150417035025009 (2015). doi:10.1056/NEJMoa1503780
8. Tibayrenc M. *Encyclopedia of Infectious Diseases*: Modern Methodologies. John Wiley & Sons; 2007.
9. Powers, W. J. *et al.* 2018 Guidelines for the Early Management of Patients With Acute Ischemic Stroke: A Guideline for Healthcare Professionals From the American Heart Association/American Stroke Association. *Stroke; a journal of cerebral circulation* **49,** e46–e110 (2018).
10. Sheth, S. A. *et al.* Time to endovascular reperfusion and degree of disability in acute stroke. *Ann Neurol* **78,** 584–593 (2015).
11. Saver, J. L. *et al.* Time to Treatment With Endovascular Thrombectomy and Outcomes From Ischemic Stroke: A Meta-analysis. *JAMA* **316,** 1279–1288 (2016).
12. Barman, A. *et al.* Determining Ischemic Stroke From CT-Angiography Imaging Using Symmetry-Sensitive Convolutional Networks. *IEEE International Symposium on Biomedical Imaging ISBI (2019) (in print)*
13. Saver, J. L. Time is brain--quantified. *Stroke; a journal of cerebral circulation* **37,** 263–266 (2006).
