# Supplementary material for: Automated Large Vessel Occlusion Detection Software and Thrombectomy Treatment Times: A Cluster Randomized Clinical Trial
Source: JAMA Neurol. 2023 Sep 18;80(11):1182–90. doi: 10.1001/jamaneurol.2023.3206 (PMC10507590; doi:10.1001/jamaneurol.2023.3206)
Supplement: Supplement 3. — Data sharing statement [file jamaneurol-e233206-s003.pdf]

Data Sharing Statement

Data
Data available: No

Additional Information
Explanation for why data not available: The data supporting the findings of this trial are available from the corresponding author upon request, following clearance from the local ethics committee. Ethical approval was obtained from the UTHealth Institutional Review Board, and the requirement for patient consent was waived (HSC-MS-19-0630). This trial was registered with clinicaltrials.gov (NCT05838456)
